# Monitoring invasive pines using remote sensing: a case study from Sri Lanka

**DOI:** 10.1007/s10661-023-10919-1

**Published:** 2023-01-31

**Authors:** W.D.K.V. Nandasena, Lars Brabyn, Silvia Serrao-Neumann

**Affiliations:** 1 Geography Programme, School of Social Sciences, University of Waikato, Hamilton, New Zealand; 2 Department of Geography and Environmental Management, Faculty of Social Sciences and Languages, Sabaragamuwa University of Sri Lanka, Belihuloya, Sri Lanka; 3 Environmental Planning Programme, School of Social Sciences, University of Waikato, Hamilton, New Zealand; 4 Cities Research Institute, Griffith University, Brisbane, Australia

**Keywords:** Google Earth Engine, Invasive pine, Landsat, *Pinus caribaea*, Random forest classifier, Sri Lanka

## Abstract

Production plantation forestry has many economic benefits but can also have negative environmental impacts such as the spreading of invasive pines to native forest habitats. Monitoring forest for the presence of invasive pines helps with the management of this issue. However, detection of vegetation change over a large time period is difficult due to changes in image quality and sensor types, and by the spectral similarity of evergreen species and frequent cloud cover in the study area. The costs of high-resolution images are also prohibitive for routine monitoring in resource-constrained countries. This research investigated the use of remote sensing to identify the spread of *Pinus caribaea* over a 21-year period (2000 to 2021) in Belihuloya, Sri Lanka, using Landsat images. It applied a range of techniques to produce cloud free images, extract vegetation features, and improve vegetation classification accuracy, followed by the use of Geographical Information System to spatially analyze the spread of invasive pines. The results showed most invading pines were found within 100 m of the pine plantations’ borders where broadleaved forests and grasslands are vulnerable to invasion. However, the extent of invasive pine had an overall decline of 4 ha over the 21 years. The study confirmed that remote sensing combined with spatial analysis are effective tools for monitoring invasive pines in countries with limited resources. This study also provides information to conservationists and forest managers to conduct strategic planning for sustainable forest management and conservation in Sri Lanka.

## Introduction

Forest plantations have been established worldwide to provide timber and firewood and have significantly contributed to local and global economies. However, most of these plantations were established using tree species with fast growth rates, which differ from many naturally regenerated forests in composition and structure, leading to different ecological processes and functional outcomes (Subasinghe et al., 2014). In some environments, these fast-growing exotic tree species have become invasive, replacing existing vegetation (Dash, 2020; Rejmánek & Richardson, 2013; Richardson et al., 2007).

Notably, plantations using pine species are the highest contributor to timber production globally (Burley & Barnes, 2004; McEwan et al., 2020). These species, however, can cause many adverse effects to the environment. For example, pine species native to North America and Europe have been introduced to New Zealand, South Africa, Argentina, Brazil, and Chile as a foundation for exotic forestry enterprises (Pauchard et al., 2016a; Rejmánek, 2014; Singh et al., 2018), but are now invading significant areas such as native forests, grasslands, catchments, and protected areas, causing a potential transformative effect on native ecosystems (Dash, 2020; Richardson et al., 2007). Additionally, Medawatte et al. (2010) study shows that invasive pines can also contribute to an overall decrease in native biodiversity, suppress native plants, contribute to habitat loss for wildlife, decrease streamflow, cause changes in nutrient cycling, and affect groundwater supply levels.

*Pinus patula* and *Pinus caribaea* were introduced to Sri Lanka in 1967 to boost timber production and reduce the risk of soil erosion and landslides in the hilly regions due to their faster growth rates compared with indigenous species (Subasinghe, 2007). In particular, *Pinus caribaea* was used extensively for the reforestation of degraded areas in the country’s wet and intermediate climatic zones with elevations ranging from 100 to 2000 m above mean sea level due to its fast establishment and growth under adverse conditions (Edirisinghe, 2017; Jayawardhane & Gunaratne, 2020; Perera, 2001). However, their invasive behavior in Sri Lanka’s mountainous region has negatively affected the soil biodiversity and regeneration of native flora, and increased the occurrence of wildfires during the dry season (Nissanka et al., 2005; Wijerathna et al., 2016). In 2008, *Pinus caribaea* was identified as a potentially invasive alien species in Sri Lanka because several areas in the mid-country reported their spread (Wijesundera, 2008). As a result, the Sri Lankan government is planning to replace pine plantations with indigenous plant species (Office of the Cabinet of Ministers-Sri Lanka, 2017).

Previous studies (Bjerreskov et al., 2021; Kaplan, 2021; Liu et al., 2022) have investigated the expansion of broad leaf and conifer forests maintained and managed for commercial timber production in temperate climates where the difference is very significant due to seasonal and phenological changes. Furthermore, it is relatively easy to recognize these two types of temperate forests when coniferous species are evergreen and broad leaf trees are deciduous. Comparatively, studies conducted in tropical environments are limited in their spatial extent and are focused on the simple classification of landscapes into major land cover classes (Petersen et al., 2016). Furthermore, Pauchard et al. (2016b) found that climate-induced invasion, or the latitudinal pattern of invasion, happened all around the world in both tropical and temperate locations showing a growing need to monitor the spread of exotic species, and forecast future distribution of such invasions. Most pinus invasion studies (Pauchard et al., 2016; Weisberg et al., 2007; Xu et al., 2018), however, have been based on temperate region and largely on grasslands and shrublands. Additionally, although there are many records of exotic pines invading the natural vegetation in tropical regions (Afrin et al., 2010; Ayala et al., 2005), few studies have been performed with the use of remote sensing techniques (Amaral et al., 2015; Goncalves et al., 2022; Petersen et al., 2016). For example, only one recent study conducted in Sri Lanka has used Geographic Information System (GIS) analysis (Medawatte et al., 2010). Mapping the expansion of invasions through field surveys is time-consuming and costly; hence, there is a need to develop remote sensing methods that are both reliable and affordable, especially in developing countries.

Remote sensing is a promising tool for mapping, detecting, and monitoring invasive non-native plants across broad geographic extents. The availability of satellite image archives permits mapping non-native invasive plants spread, retrospectively (Gavier-Pizarro et al., 2012). However, most existing studies (Amaral et al., 2015; Andrew & Ustin, 2008; Dash et al., 2019; Khare et al., 2018; Piiroinen et al., 2018) have used high-resolution hyperspectral imagery covering a relatively small area and analyses over a short period of time. From a management perspective, this limits their use for long-term assessments of invasive plant spread, particularly in developing countries where the lack of historical data further compounds management efforts. On the other hand, multispectral sensors with moderate resolutions offer an alternative for low-resourced countries because they enable both change detection and measurements of phenology over multiple years (Petersen et al., 2016; Signori & Ducati, 2019; Xu et al., 2018). Additionally, these data sets are mostly free or low cost (Bradley, 2014). In particular, the Landsat program has been extensively used for forest monitoring, providing a rich dataset to map the invasion of tree species at the landscape scale. The opening of the Landsat archive and its continued free and open access since 2009 has enabled the analysis of both larger areas and more extensive times series (Zhu & Woodcock, 2014).

The current study investigates the application of Landsat satellite information to map, quantify and assess spatio-temporal land cover change using a Sri Lankan study area, namely Belihuloya, over a two-decade period (2000 to 2021). The selected study area is one of the few areas that manage pines for both conservation and commercial purposes in Sri Lanka. The Belihuloya region suffers from a shortage of groundwater caused by the water uptake from pine plantations (Starkloff, 1998). This results in seasonal wildfires, so it is therefore important that pine forest expansion is monitored. This study contributes to knowledge development by analyzing the spread of *Pinus caribaea* to neighboring landscapes over a 20-year period from plantations established and operated for soil conservation in Sri Lanka. Specifically, the paper seeks to answer the following research question: to what extend is the Landsat archive for Sri Lanka suitable for mapping and tracking land cover changes associated with the historical spread and invasion of conifers? To this end, this study uses Landsat archive to distinguish unmanaged pine plantations from native forests in Sri Lanka’s tropical environment.

## Materials and methods

### Study area

**Fig. 1:**
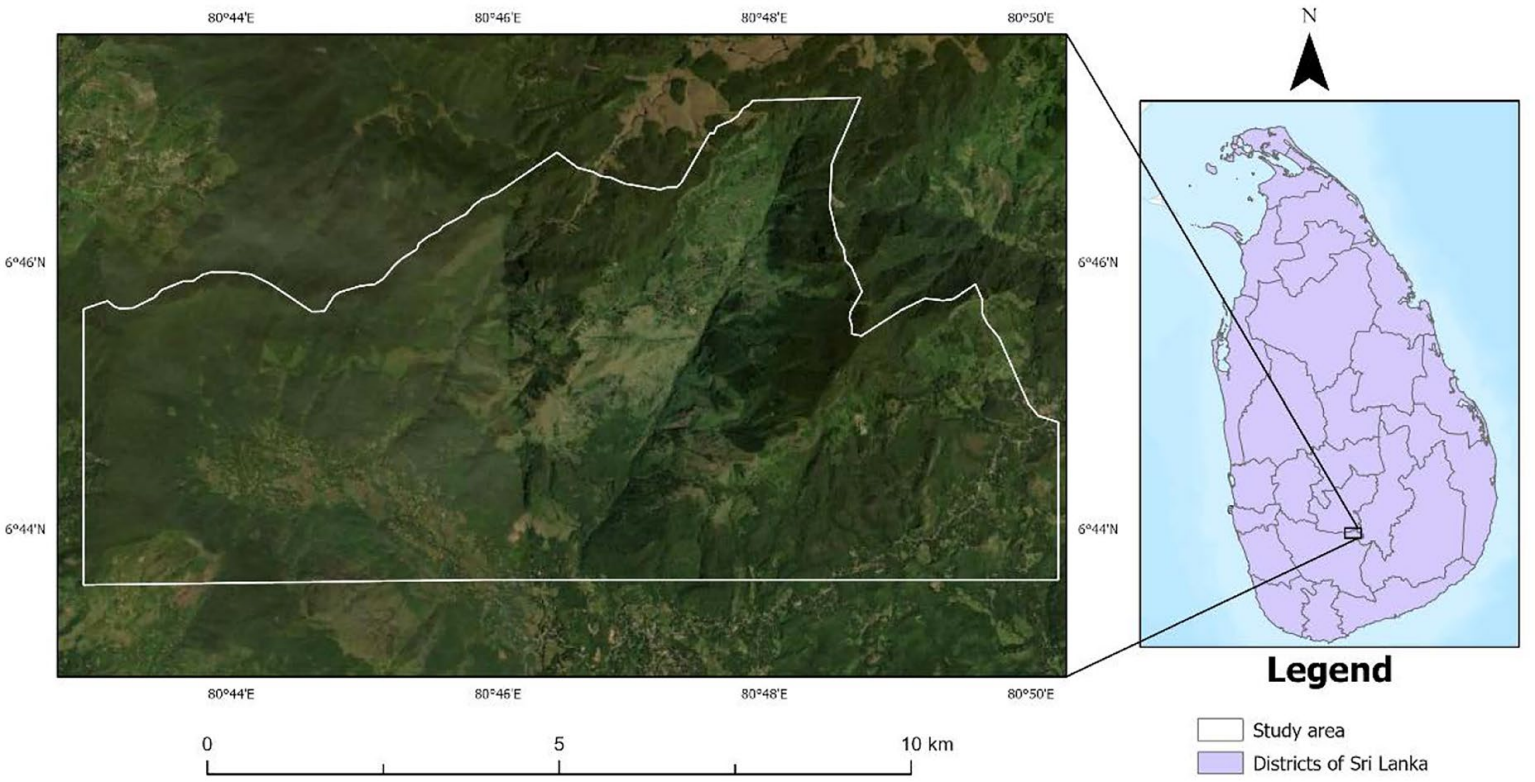
Study area

This study used images from the Landsat archive for the years 2000 and 2021 to monitor the historical spread and control of invasive exotic conifers (e.g., *Pinus caribaea*) in Sri Lanka’s intermediate climatic zone. Sri Lanka has traditionally been generalized into three climatic zones: the wet zone, dry zone, and intermediate zone, according to rainfall, soil, and vegetation types. The intermediate zone separates both the wet and dry zones and receives a mean annual rainfall between 1750 and 2500 mm with a short and less prominent dry season (Punyawardena, 2020). This intermediate climatic zone divides into three further zones based on altitude: low-country, mid-country, and up-country. According to the Sri Lankan climatic classification (Jayawardane & Weerasena, 2000), this study area lies in the intermediate mid-country, where the elevation ranges between 600 and 900 m above mean sea level and contains rugged topography. The study area is located in Southeast Sri Lanka and covers an area of 6234 ha. Administratively, this area belongs to the Balangoda region in the Sabaragamuwa province of Sri Lanka (Fig. 1). The area is densely forested, containing a mixture of forest types, including montane broadleaf forest, pine plantations, and grasslands. This area was selected because it comprises both unmanaged pineland and densely forested native broad leaf species.

### Methods

**Fig. 2:**
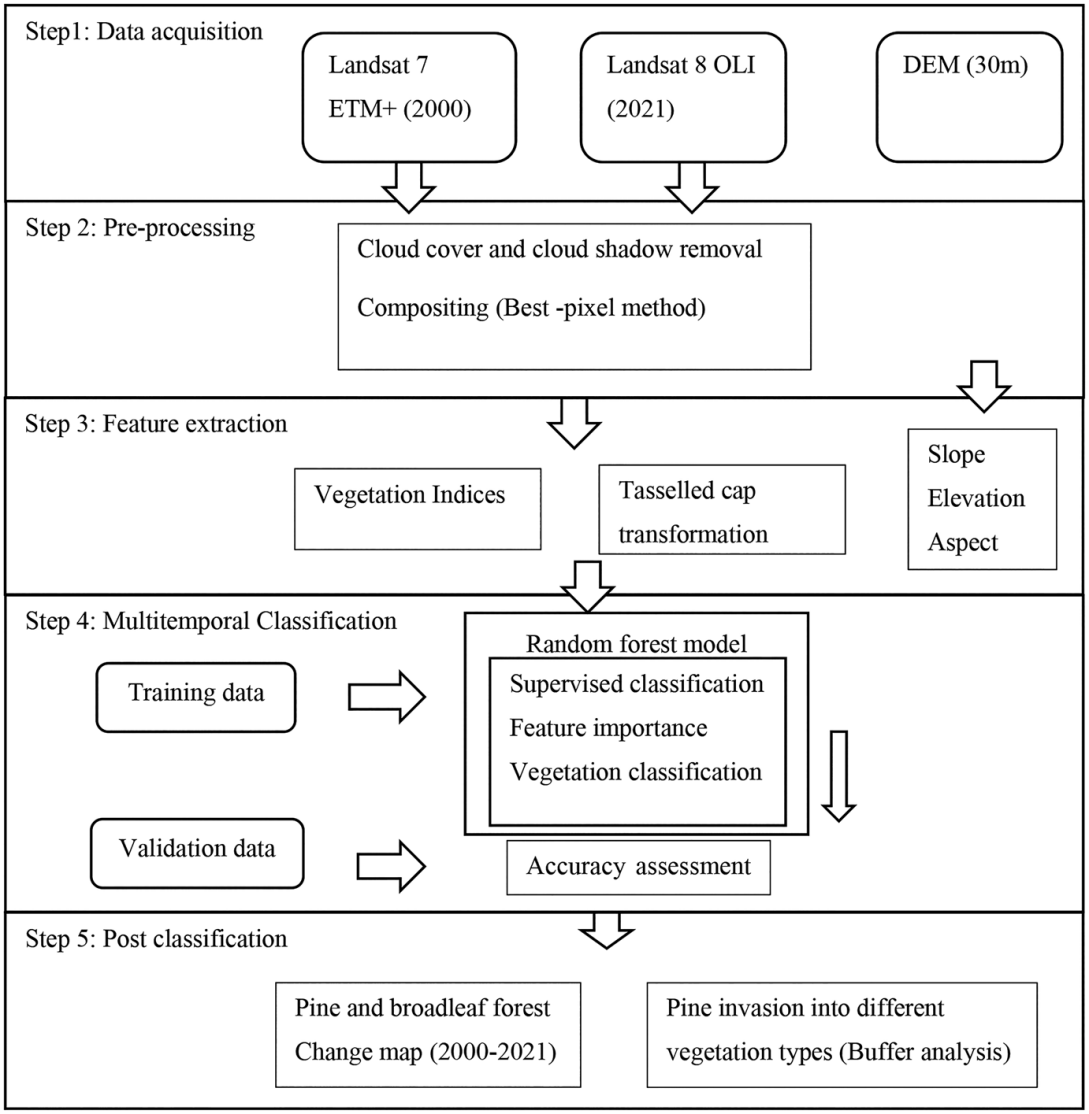
Overall methodology

The following section describes the methodology developed for this research. As shown in Fig. 2, the study comprised five main steps: (1) data acquisition; (2) pre-processing; (3) feature extraction; (4) multitemporal classification; and (5) post-classification.

#### Step 1: Data acquisition

Landsat 7 Enhanced Thematic Mapper Plus (ETM +) and Landsat 8 Operational Land Imager (OLI) images from the Google Earth Engine (GEE) Landsat archive (https://developers.google.com/earth-engine/datasets/catalog/landsat) for the years 2000 and 2021 were used to characterize vegetation changes. The advantage of Landsat as a moderate spatial resolution satellite collection is that the entire scene is captured at once with sun illumination, vegetative condition, and atmospheric conditions more likely to be consistent across the scene (Labonté et al., 2020). The study period 2000 and 2021 was chosen as it is difficult to validate the classification prior to 2000 due to the absence of high-resolution images in Google Earth Pro. In 2000, only six images of Landsat 7 that covered the study area were available in the Landsat archive, and in 2021, 21 images of Landsat 8 were available. The GEE cloud-based platform was used for image pre-processing and classification (Setiani et al., 2021), while ArcGIS Pro software was used to produce maps.

The Shuttle Radar Topographic Mission (SRTM) provides digital elevation data (DEM) on a global scale. The SRTM Plus version (spatial resolution of 30 m) was acquired from GEE, and topographic features such as elevation, aspect, and slope were extracted.

#### Step 2: Pre-processing

As the studied region is frequently cloudy, it is difficult to acquire cloud-free Landsat images. Therefore, atmospheric correction, cloud/shadow masking, and image compositing were applied to construct a clean image time series. A cloud masking procedure was used to identify flagged cloud and cirrus pixels. After cloud removal, gap filling was applied using the median values of each pixel on the images. Then the median composite function in GEE was used to remove anomalous dark pixels (shadows) and bright, saturated pixels (Bjerreskov et al., 2021) in the collection and was used to create composite images for the years 2000 and 2021.

#### Step 3: Feature extraction

Feature extraction was carried out in GEE using vegetation indices derived from spectral data and tasselled cap transformation, which transforms spectral data into indicators (Dash, 2020).

##### Vegetation indices

Throughout all wavelengths, coniferous leaves have lower transmittance than broad leaves (Lukeš et al., 2013). Though the difference between visible (VIS) and near-infrared (NIR) spectral reflectance is not hugely evident, some studies (Roberts et al., 2004; Williams, 1991) have reported that coniferous needles have slightly lower VIS reflectance and higher NIR reflectance than broadleaves. It is hard to classify land cover or identify specific species when the vegetation types are heterogeneous and often exhibit both spectral and seasonal similarities (Deng et al., 2020). Therefore, several vegetation indexes were used as shown in Table 1. These were as follows: (1) normalized difference moisture index (NDMI); (2) normalized burn ratio (NBR); (3) enhanced vegetation index (EVI); (4) normalized difference vegetation index (NDVI); and (5) green chlorophyll index (GCI). These indices categorized vegetation changes related to moisture stress, colorization, and needle/leaf structure (Ye et al., 2021).

**Table 1:** Bands and vegetation indexes

**Index**	**Equation**	**Source**
NDMI	$\frac{\left(NIR-SWIR1\right)}{\left(NIR+SWIR1\right)}$	Shahfahad et al. (2022)
NBR	$\frac{(NIR-SWIR)}{(NIR+SWIR2)}$	Setiani et al. (2021)
EVI	$2.5\times\frac{NIR-RED}{(NIR+6\times RED-7.5\times BLUE+1)}$	Senf et al. (2013)
NDVI	$\frac{\left(NIR-RED\right)}{\left(NIR+RED\right)}$	Pu et al. (2008)
GCI	$\left(\frac{NIR}{GREEN}\right)-1$	Kumar et al. (2018)

##### Tasselled cap transformation

Tasselled cap transformation (TCA) was developed by Kauth and Thomas (1976) as a function for determining a crop’s life cycle. It recognizes the pattern found in the agricultural fields where there is a net increase in near-infrared and decrease in red reflectance based on soil color.

The tasselled cap transformation is a linear transformation of Landsat MSS data that projects soil and vegetation information into a single plane in a multispectral data space. TCA has been broadly engaged in forestry studies of structure, condition, successional state, and change detection in various forest environments (Gómez et al., 2012). It is a special case of principal components analysis which transforms the image data into a new coordinate system with a new set of orthogonal axes. The tasselled cap analysis reduces a multiband data set (4–6) to 3 channels as follows:

- Tasselled cap greenness (TCG) contrasts the near-infrared and visible bands, conveying information concerning the abundance and vigor of living vegetation.
- Tasselled cap brightness (TCB) is a weighted sum of all four bands. Brightness is defined in the direction of the principal variation in soil reflectance and is associated with bare areas or partially covered soil.
- Tasselled cap wetness (TCW) is related to the canopy and soil moisture which differences the sum of the visible and near-infrared bands with the longer infrared bands.

#### Step 4: Multitemporal classification

The supervised classification of both maps (the years 2000 and 2021) was carried out using the Random Forest (RF) classifier because of its relatively high accuracy and computational efficiency (Zhu & Woodcock, 2014). Random Forest is an integrated learning method that has become increasingly common in remote sensing applications due to its nonparametric nature and ability to limit overfitting (Cheng & Wang, 2019). The Smile Random Forest function on the GEE platform (Liu et al., 2022) was used, and the number of decision trees was set to 500. A score was calculated using the RF algorithm for each combination of parameters. The combination of parameters with the highest score was used for developing the vegetation classification maps.

##### Training and validation

Stratified random sampling was used to obtain a sample population that best represents each landcover class. In these samples, 20 polygons for each class were randomly identified by the visual interpretation of the Landsat images with the help of Google Earth (Mohamed & El-Raey, 2019; Zhu & Liu, 2014). Then, five hundred training samples per class were generated to train the classification for each year.

The goal of the validation was to assess the overall accuracy of the vegetation change map compared to available reference data. Validation was based on existing datasets and a combination of ancillary sources (Google earth, field plots) assembled using a human interpreter approach (Weng, 2018). High spatial resolution images from Google Earth Pro were used to manually interpret the land cover classes and also help determine land cover change at longer intervals (Kamga et al., 2020; Mohamed & El-Raey, 2019; Zhu & Woodcock, 2014). This research used high-resolution images of 2021 and 2000 in Google Earth pro as reference data. Specifically, about 120 samples were used as ground reference data for each landcover class to train the random forest classifier except for the settlement class, which was smaller in 2000. The classification accuracy was tested using the overall agreement with reference data. In addition, the user and producer agreements for the five landcover types were calculated. The producer accuracy measures the percentage of given reference data correctly classified; in contrast, the user accuracy measures the percentage of the trained data correctly classified (April et al., 2015).

#### Step 5: Post-classification

Following the classification of imagery for the two individual years, a post-classification approach of subtracting the classification maps was applied and used to produce a detailed change detection map using the ArcGIS Pro software. This map detects more subtle classes of change and determines the invasive pine and its spread over the selected landscape. An important aspect of change detection is determining what is changing to what category of land use type (Abebe et al., 2021). Therefore, a vegetation conversion matrix was calculated to demonstrate the direction of change and the land use type that remains at the end of the study period. Quantitative data analysis of the overall land cover change as well as gains and losses in each category between 2000 and 2021 were compiled (Haregeweyn et al., 2013; Kamga et al., 2020) and presented as a percentage of the area. In this manner, maps were created for the entire study area and provided insight into the areas affected by invasive conifer encroachment.

##### Buffer analysis: identification of invasive pine

In order to investigate the progressive spread of *Pinus caribaea* outside the original plantations, the distance from the perimeter of parental pine plantations was measured using six 50-m intervals between 0 and 300 m demarcated. The buffer distance was based on the previous study by Medawatte et al. (2010) in Sri Lanka. The area of invading pines was measured in relation to the distance from the plantation edge.

## Results

**Fig. 3:**
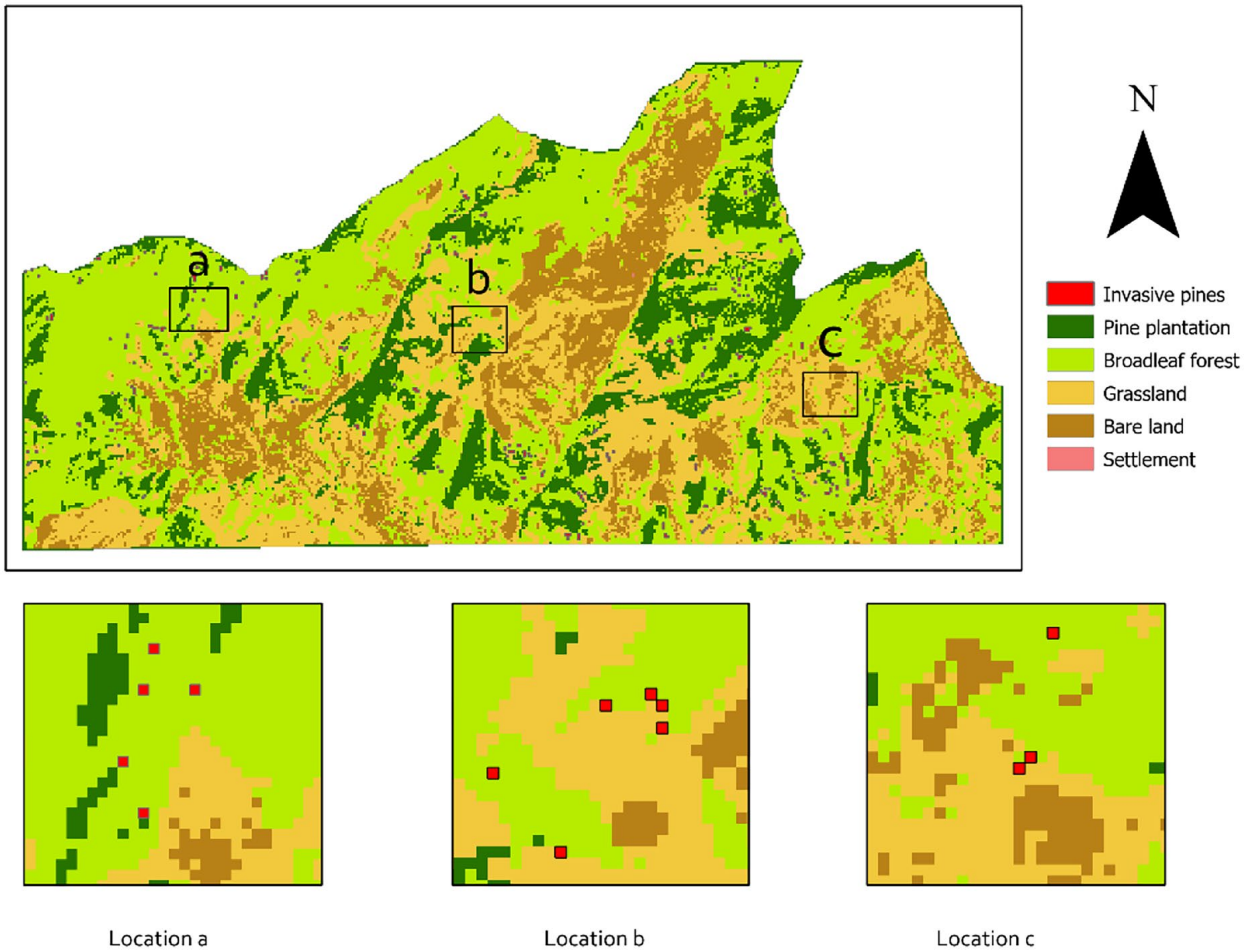
Vegetation classification for the year 2000

**Fig. 4:**
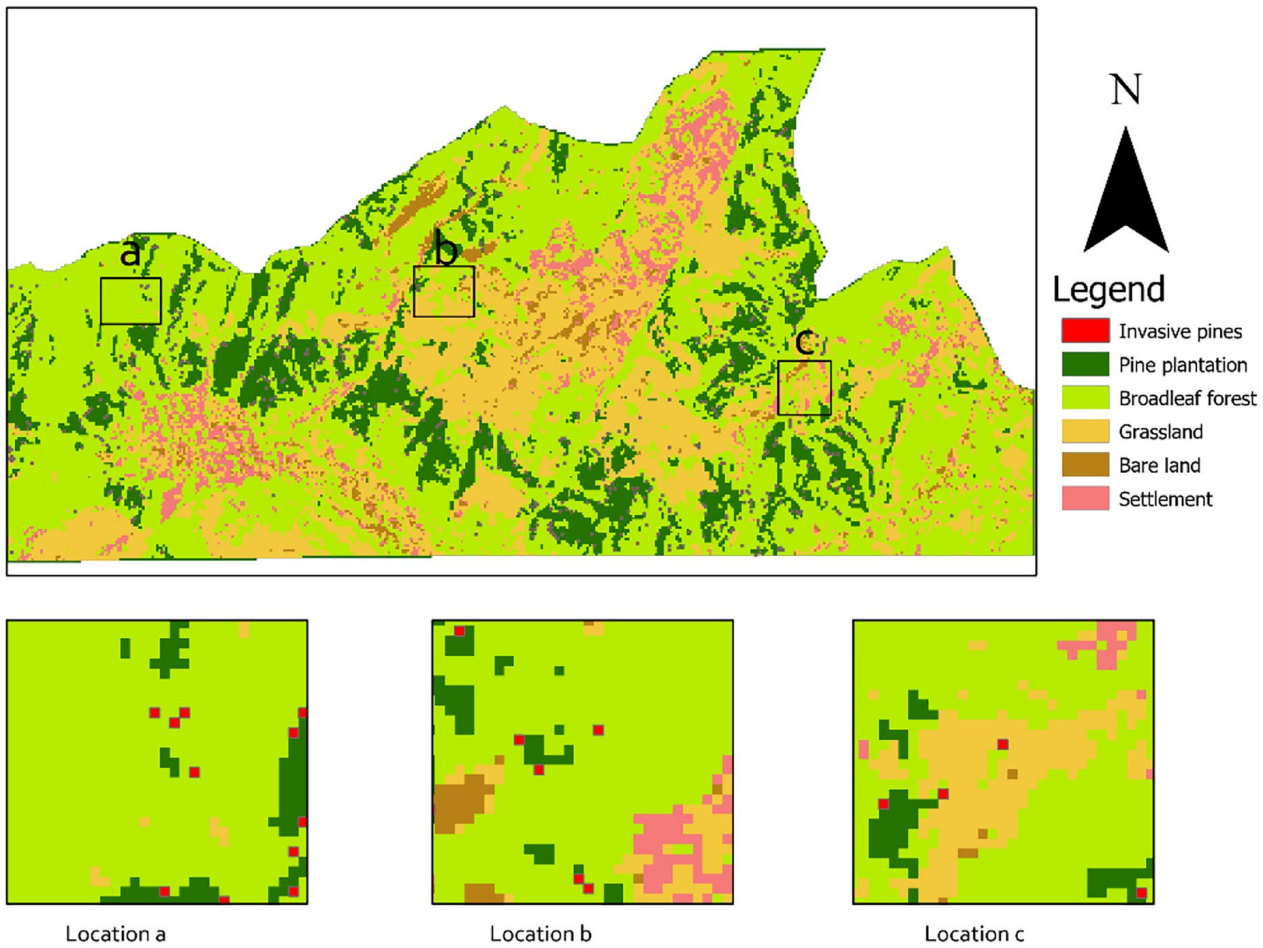
Vegetation classification for the year 2021

The vegetation maps for 2000 and 2021 are shown in Fig. 3 and Fig. 4, respectively. These maps collectively show places with stable and changed vegetation areas appearing in the study area. Five major vegetation types were classified for 2000 and 2021: pine plantation, broadleaf forest, bare land, grassland, and invasive pines (settlements are also identified). The most prominent vegetation type found in both maps (2000 and 2021) is the broadleaf forest which is mainly distributed on the gentle slopes and the flat regions of the study area. In contrast, pine plantations and grasslands are located on ridges and steep areas with a slope higher than 18%. The vegetation classification for the year 2000 image shows that the majority of the study area was under broadleaf forest, covering about 2690 ha (43%). Grassland and bare land cover an area of 1627 ha (26%) and 969 ha (16%), respectively. Broadleaf forest (3336 ha) still covered the largest area in 2021, which reflects the conversion of other classes to the natural forest for conservation purposes, while grassland, pine plantation, and bare land cover an area of 1284 ha, 1013 ha, and 244 ha, respectively. The land areas contain pine species outside the plantation boundaries, with an absence of planting patterns, and areas smaller than 0.5 ha are identified as invasive pines.

Overall agreement with reference data was computed for both vegetation maps. The year 2000 vegetation map produced a 72% overall accuracy, and the year 2021 vegetation map yielded an 81% overall accuracy. Vegetation groups were classified with producer accuracy ranging from 20–84 and 55–100 for the year 2000 and 2021, respectively. In the year 2000 map, the settlement had the highest user accuracy (Appendix 1). On the other hand, pine plantation had the highest user accuracy for the year 2021, whereas all other vegetation classes have more than 70% user accuracy except the grassland class (Appendix 2).

The cover and rate of changes in each vegetation type for the 21 years are summarized in Table 2. From 2000 to 2021, the broadleaf forest and pine plantation area increased by 24% and 19%, respectively. In contrast, bare land and grassland showed a reverse trend, reduced by 75% and 21%, respectively, during the same period. This indicates that the increasing broadleaf forest area mainly emerged in the steep slope areas, meaning some grassland and bare land areas were converted to natural forests without disturbance.

**Table 2:** Extent and rate of vegetation changes between 2000 and 2021

Vegetation type	2000		2021		Change between 2000 and 2021	
	Area (ha)	%	Area (ha)	%	Area (ha)	%
Invasive pine	93	1	89	0	− 4	− 4
Pine plantation	854	14	1013	16	159	19
Broadleaf forest	2691	43	3336	54	646	24
Grassland	1627	26	1284	21	− 343	− 21
Bare land	969	16	244	4	− 725	− 75
Settlement	0.18	0	269	4	268.82	

The vegetation conversion matrix depicts the direction of change and the land use type that remains at the end of the period. Thus, the change matrix for each period was analyzed to understand the source and destination of significant vegetation changes. Over 21 years, 3476 ha out of 6234 ha of the total area has not changed, accounting for 56% of the study area (Table 3). In 2021, there was a significant settlement build up activity, with an area of 269 ha or 4% of the region’s total area (Table 2).

**Table 3:**
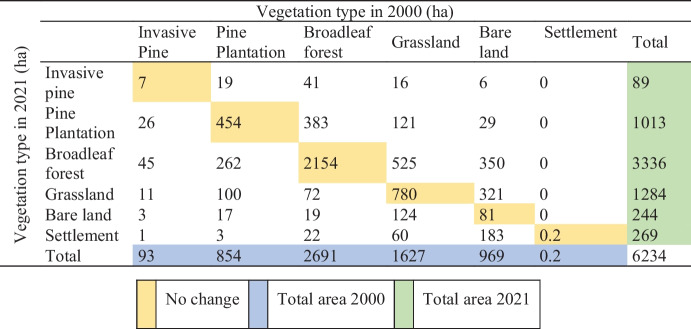
Vegetation conversion matrix between 2000 and 2021

Significantly, from 2000 to 2020, approximately 705 ha of bare land and 663 ha of grassland have converted to other vegetated areas. The increase in settlement surfaces is coupled with the decline in these grasslands and bare land areas. Furthermore, approximately 80% of the area covered with broadleaf forest in 2000 was still the same in 2021. The remaining 20% (536 ha) was transformed to other uses in 2021. Of the total pine plantation cover in 2000, 53% remained unchanged, while the remaining portion vastly changed to grassland (100 ha) and broadleaf forest (262 ha). Overall, there was a significant increase in the broadleaf forest from 2691 to 3336 ha. This gradual increase is attributed to the replanting programs carried out in the western part of the study area (Kumara, 2010).

The invasive pine areas during this period showed a 4% slight decline. Concurrently, 45 ha of invasive pine areas was converted to broadleaf forest, and 15 ha was cleared. Furthermore, 26 ha of invasive pine in 2000 was identified as pine plantations in 2021, whereas 19 ha of pine plantations in 2000 was recognized as invasive pine in 2021. This misperception may be due to less aerial visibility of continuous clumps of pine cover over the area in 2000, with an identifiable canopy only appearing later.

**Fig. 5:**
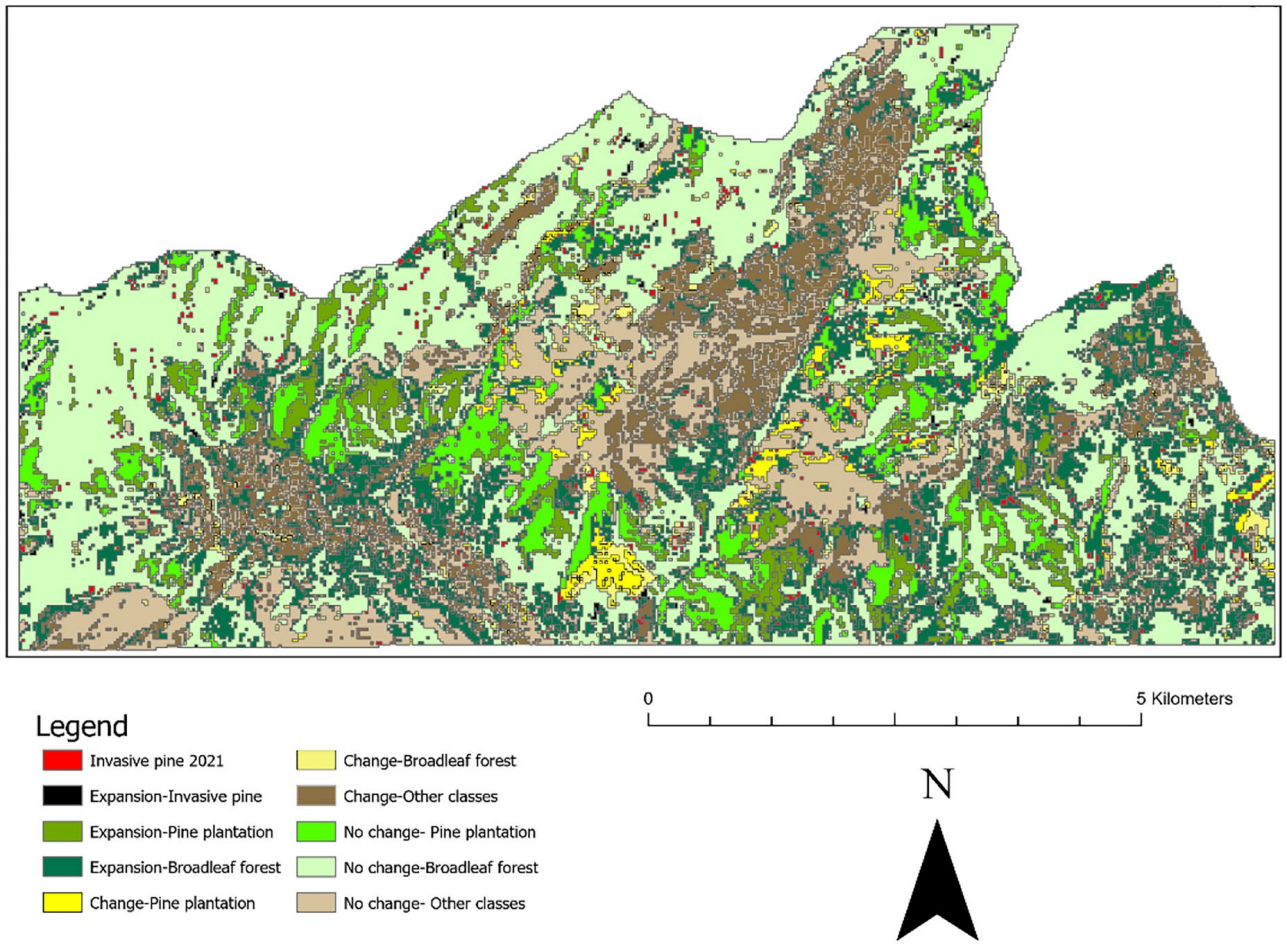
Pine and broadleaf forest change map 2000–2021

As this study focuses on the dispersal of invasive pine around the study area, the following section emphasizes the extent of escaped pines from the pine plantations into the adjacent habitats. Figure 5 observes the conversion of pine plantations and broadleaf forests from 2000 to 2021. Moreover, an expansion of pine plantations was detected primarily around the existing plantations of 2000. Broadleaf forests in more remote valley areas tended to retain extensive forest in 2021, and broadleaf forest expansion or regeneration of the forest can be observed primarily on grassland. During the 21 years, pine plantations have increased by 159 ha, whereas invasive pine declined by 4 ha. Thus, invasive pines are more visible along with broad leaf forests managing the same borders as the pine plantations.

**Fig. 6:**
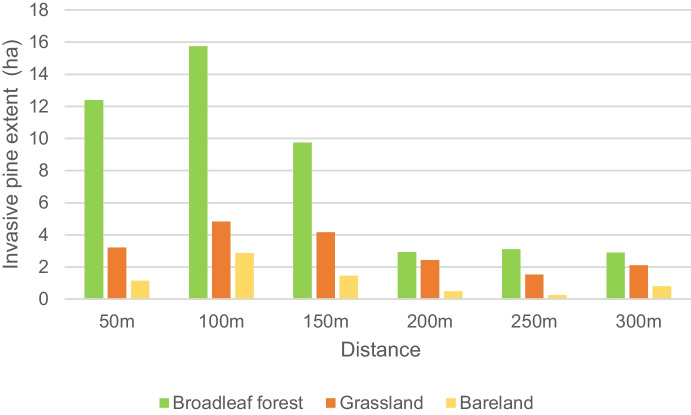
Invasive pine expansion into the different vegetation types adjacent to pine plantations

The extent of invading pines was measured with respect to the distance from the plantation edge. For the buffer analysis, three different vegetation types were selected: broadleaf forest, grassland, and bare land, which are adjacent to the pine plantations. The results showed that pines invaded broadleaf forests to a greater extent than the other two habitat types (Fig. 6). Furthermore, analysis showed that the highest scattering of pines was within 100-m buffer zone of the plantation, while encroachment of pines reduced beyond the 100-m buffer zone.

## Discussion

This research demonstrated that freely available Landsat images combined with topographical data can be used for mapping vegetation change over a twenty-year time period, confirming its applicability for mapping and understanding the extent of pine invasion. The results have revealed that pine invasion is at a moderate level but is decreasing might be due to anthropogenic activities.

The research has found that a combination of spectral, textural, and topographical bands provides an effective solution for classifying the vegetation cover, similar to the findings of Yu et al. (2020) on forest classification in subtropical regions, and the discriminating of urban forest types in a semi-humid monsoon region in China by Zhou et al. (2019). Earlier works by Wang et al. (2006) and Zhu and Liu (2014) had specified that using topographical data with spectral data improved the discrimination between conifers and broadleaf forests. Correspondingly, studies by Peterson (2005) and April et al. (2015) had found that model accuracy was slightly better using topographical parameters. In contrast, Bjerreskov et al. (2021) found that the elevation did not improve accuracy when classifying broadleaf and conifer forest in the Danish landscape. However, it appears that elevation plays a big role in forest classification in mountain terrain or when the landscape is complex, as demonstrated by this research.

This study obtained accuracies for the vegetation cover classification similar to those obtained by other studies using multi-year Landsat images (Coyle et al., 2020; Gómez et al., 2012), and the accuracies obtained for land classification using other higher resolution satellite images (Hantson et al., 2012; Perera, 2001). The classification accuracy of forest types obtained in this study is higher that obtained studies by Akumu et al. (2021), Senf et al. (2013), and an investigation by Sá et al. (2017), which focuses on the invasion of *Acacia longifolia* in a pine forest. However, studies by Andrew and Ustin (2008); Bjerreskov et al. (2021); Fagan et al. (2018); Hestir et al. (2008); and Underwood et al. (2003) obtained higher classification accuracies than the present study by using satellite imagery with a higher resolution.

Even though better results can be obtained with higher resolution images (Goncalves et al., 2022), Landsat has been shown to provide long-term assessments of invasive plant spread (Gavier-Pizarro et al., 2012; Labonté et al., 2020; Sá et al., 2017). Furthermore, the freely available Landsat series of satellites is one of the most important data sources for developing countries that have budget constraints (Gavier-Pizarro et al., 2012; Haregeweyn et al., 2013). Liu et al. (2022) concluded that Landsat is better for detecting long-term changes in Chinese pine tree species because of its availability over a long time period. For developing countries such as Sri Lanka, we conclude that Landsat images provide a vital low-cost solution for the frequent monitoring of vegetation change.

According to this study’s findings, broadleaved forests and grasslands are vulnerable to invasion. Previous researchers (Dash et al., 2019; Higgins & Richardson, 1998; Medawatte et al., 2010) found that grasslands were more prone to invasion than other areas. However, Goncalves et al. (2022), Förster et al. (2017), and Ayala et al. (2005) identified forest habitats as the most vulnerable to the pine invasion. Forest habitats become vulnerable to pine invasion when exposed to disturbances such as wildfire (Medawatte et al., 2010). Moreover, most invading pines were found within a 100-m buffer zone for all types of habitats, while encroachment declined sharply beyond the 50–100-m buffer zone. These results are in line with those of Ayala et al. (2005).

An important finding in this research was the decline in invasive pine in the Belihuloya region. Wijesundera (2008) had stated that *Pinus caribaea* had spread rapidly in several mid country areas and predicted that it could be a potential invasive alien in the near future. Due to social pressure from local communities, pine planting was stopped (Edirisinghe, 2017), and the country has identified pines an invasive species (Medawatte et al., 2010). In 2017, the Sri Lankan government implemented a project to plant indigenous plant species to develop an under-storey in the existing pine plantations (Office of the Cabinet of Ministers-Sri Lanka, 2017). This research gives insight into the invasion of pines in the intermediate zone and helps inform management strategies to maintain this downward trend.

While the study could have obtained better classification accuracy using higher spatial or spectral resolution images, these are not free and are only available for recent years. Extending the study period to before 2000 could also have reduced its limitation, but it would have been difficult to validate a classification prior to 2000 due to the absence of high-resolution images in Google Earth Pro.

Future research could seek to understand the spatial variation in vegetation change and the reasons the invasive pines spread to certain areas. For example, the relationship between pine invasion and disturbances such as forest fires could be explored. Areas impacted by forest fire could be remotely sensed using satellite images. Furthermore, mapping and monitoring invasive pines in other climatic regions would also be relevant for forest management and conservation planning.

## Conclusion

This study demonstrated the extent of pine invasion in the Belihuloya region, Sri Lanka. Remote sensing has enabled the mapping of vegetation change in the study area between 2000 and 2021 using freely available Landsat data. A combination of spectral, textural, and topographical bands provided an effective solution for detecting the spread of invasive *Pinus caribaea*. This study provided evidence that pines have invaded the natural habitat in the intermediate climate areas of Sri Lanka, especially native forests, and grasslands but the extent of this invasion has declined between 2000 and 2021.

It is clear that remote sensing is a valuable tool for monitoring and understanding forest dynamics, and the use of freely available satellite images, such as Landsat, is particularly important for developing countries such as Sri Lanka. The availability of the Google Earth Engine platform and its extensive range of analysis functions and free processing power is also very beneficial for countries with limited financial resources.

## Data Availability

The data that support the findings of this study are openly available in GEE at https://code.earthengine.google.com/5a840aa5fb12eba70dc0802153319af9.

## Appendix 1 Confusion matrix for the year 2000

**Table:**

	Classification						
	Land cover	Pine plantation	Broadleaf forest	Grassland	Bare land	Settlement	Total
Reference	Pine plantation	90	14	12	4	0	120
	Broadleaf forest	0	96	24	0	0	120
	Grassland	13	3	87	17	0	120
	Bare land	6	5	30	79	0	120
	Settlement	0	4	4	8	4	20
	Total	109	122	157	108	4	500
	User accuracy (%)	82	79	58	73	100	
	Producer accuracy (%)	75	84	72	66	20	
	Overall accuracy (%)	72					

## Appendix 2 Confusion matrix for the year 2021

**Table:**

	Classification						
	Land cover	Pine plantation	Broadleaf forest	Grassland	Bare land	Settlement	Total
Reference	Pine plantation	116	4	0	0	0	120
	Broadleaf forest	0	120	0	0	0	120
	Grassland	0	4	104	12	0	120
	Bare land	0	0	30	84	6	120
	Settlement	0	18	18	18	66	120
	Total	116	146	152	114	72	600
	User accuracy (%)	100	82	68	74	92	
	Producer accuracy (%)	96	100	86	70	55	
	Overall accuracy (%)	81					
